# The Anti-Melanoma Activity of Dinaciclib, a Cyclin-Dependent Kinase Inhibitor, Is Dependent on p53 Signaling

**DOI:** 10.1371/journal.pone.0059588

**Published:** 2013-03-18

**Authors:** Brijal M. Desai, Jessie Villanueva, Thierry-Thien K. Nguyen, Mercedes Lioni, Min Xiao, Jun Kong, Clemens Krepler, Adina Vultur, Keith T. Flaherty, Katherine L. Nathanson, Keiran S. M. Smalley, Meenhard Herlyn

**Affiliations:** 1 The Wistar Institute, Philadelphia, Pennsylvania, United States of America; 2 Massachusetts General Hospital, Boston, Massachusetts, United States of America; 3 Department of Medicine, University of Pennsylvania School of Medicine, Philadelphia, Pennsylvania, United States of America; 4 Department of Molecular Oncology, The Moffitt Cancer Center and Research Institute, Tampa, Florida, United States of America; University of Illinois at Chicago, United States of America

## Abstract

Although cyclin dependent kinase (CDK)-2 is known to be dispensable for the growth of most tumors, it is thought to be important for the proliferation of melanoma cells, where its expression is controlled by the melanocyte-lineage specific transcription factor MITF. Treatment of a panel of melanoma cells with the CDK inhibitor dinaciclib led to a concentration-dependent inhibition of growth under both 2D adherent and 3D organotypic cell culture conditions. Dinaciclib targeted melanoma cell lines regardless of cdk2 or MITF levels. Inhibition of growth was associated with a rapid induction of G2/M cell arrest and apoptosis. Treatment of human melanoma mouse xenografts with dinaciclib led to tumor regression associated with reduced retinoblastoma protein phosphorylation and Bcl-2 expression. Further mechanistic studies revealed that dinaciclib induces p53 expression whilst simultaneously downregulating the expression of the anti-apoptotic factors Mcl-1 and XIAP. To clarify the role of p53 activation in the dinaciclib-induced cell death, we generated melanoma cell lines in which p53 expression was knocked down using a shRNA lentiviral vector. Knockdown of p53 completely abolished the induction of apoptosis seen following dinaciclib treatment as shown by a lack of annexin-V staining and caspase-3 cleavage. Altogether, these data show that dinaciclib induces apoptosis in a large panel of melanoma cell lines through a mechanism requiring p53 expression.

## Introduction

The landscape of melanoma therapy has changed dramatically within the last few years with the discovery that the majority of melanomas harbor activating mutations in *BRAF* [1], [2] and that virtually all melanomas exhibit constitutive activity in the MAP kinase pathway [3]. Recently, two novel drugs were approved for the treatment of metastatic melanoma and several more are in late stage clinical development [4]. In the class of small molecule kinase inhibitors, the BRAF inhibitor vemurafenib has shown unprecedented clinical activity; but unfortunately, patients eventually develop resistance [5]. The mechanisms leading to this are under intense scrutiny and as shown by recent publications, melanoma cells can escape by various means [4], [6], [7], [8], [9]. The field is thus increasingly accepting the notion that novel targets outside of the MAPK pathway will be necessary to avoid emergence of resistant cell subpopulations and that combinations of inhibitors are the most likely to provide long lasting results [10]. Increased efforts are thus now focused on finding agents that are able to induce melanoma cell death and that may be advantageous in combination therapies with inhibitors of the MAPK pathway. Additionally, there are 50% of melanoma patients whose tumors do not harbor BRAF mutations and for whom no effective therapies are currently available.

One intriguing aspect of melanoma cells is that although they generally lack p53 mutations, they are poor at undergoing p53-dependent apoptosis [11]. Recent studies have demonstrated that p53 transcriptional targets are aberrantly expressed in melanoma; therefore reactivation of p53 could restore the proliferative balance and resistance to apoptosis in melanoma ([12]. In fact, pre-clinical studies from our laboratory and others have indicated that, p53 can be activated in melanoma cell lines, leading to the induction of apoptosis; thus the p53-dependent signaling system is not entirely dysfunctional [13], [14]. One family of compounds that could induce p53-dependent apoptosis are the cyclin dependent kinase (CDK) inhibitors [15]. CDKs are a family of serine threonine kinases that regulate both cell cycle progression and transcription through the phosphorylation of RNA polymerase II [15], [16], [17].

Cancer cells often have deregulated cell cycle regulatory mechanisms. One of the most critical steps in cell cycle entry is the phosphorylation and inactivation of the retinoblastoma (RB) protein by CDK4/6 and CDK2 [18], leading to the dissociation of the RB protein and the E2F family of transcription factors. Increased E2F activity induces transcription of EF2-dependent genes such as cyclin A, dihydrofolate reductase, thymidine kinase, and DNA polymerase-α, and promotes S-phase [19], [20], [21]. A second group of CDKs do not regulate the cell cycle but rather control RNA stability and transcription. This second family of CDKs include complexes such as cyclin H-CDK7, which phosphorylates the carboxy terminal domain of RNA polymerase II, and cyclin T-CDK9, which promote the initiation and elongation of RNA transcripts [22], [23]. Pre-clinical studies have shown that inhibition of CDK9, using flavopiridol, can reduce the expression of short-lived RNA transcripts that encode for anti-apoptotic proteins leading to cell death [15].

Among the CDK family, CDK2 has a unique role in the development and progression of melanoma. A recent study showed that CDK2 expression was regulated in melanoma cells by the melanocyte specific transcription factor MITF [24], and that CDK2 knockdown significantly reduced melanoma growth. It was further demonstrated that melanoma cell lines with lower expression of CDK2 were more sensitive to the cyclin-dependent inhibitor roscovitine [24]. These studies led to the hypothesis that CDK2 could be a useful melanoma therapeutic target.

In the present study, we have evaluated the anti-melanoma activity of a CDK1/2/5/9 inhibitor, dinaciclib, and demonstrated that the compound has broad-spectrum anti-proliferative and p53-dependent pro-apoptotic activity against a panel of melanoma cell lines.

## Materials and Methods

### Ethics Statement

This study was carried out in strict accordance with the recommendations in the Guide for the Care and Use of Laboratory Animals of the National Institutes of Health. The protocol was approved by the Wistar Institute Animal Care and Use Committee (Protocol Number: 111954). All surgery was performed under sodium pentobarbital anesthesia, and all efforts were made to minimize animal suffering.

### Cell Culture

Melanoma cells were cultured as described in [25]. The lentiviral vector short hairpin RNA (shRNA) constructs for p53 were developed in the laboratory of Dr. Maria Soengas, based on published sequences [13], [26]. Pools of p53-shRNA transduced cells were utilized in this study.

### Expression/mutational Profiling

**Figure 1:**
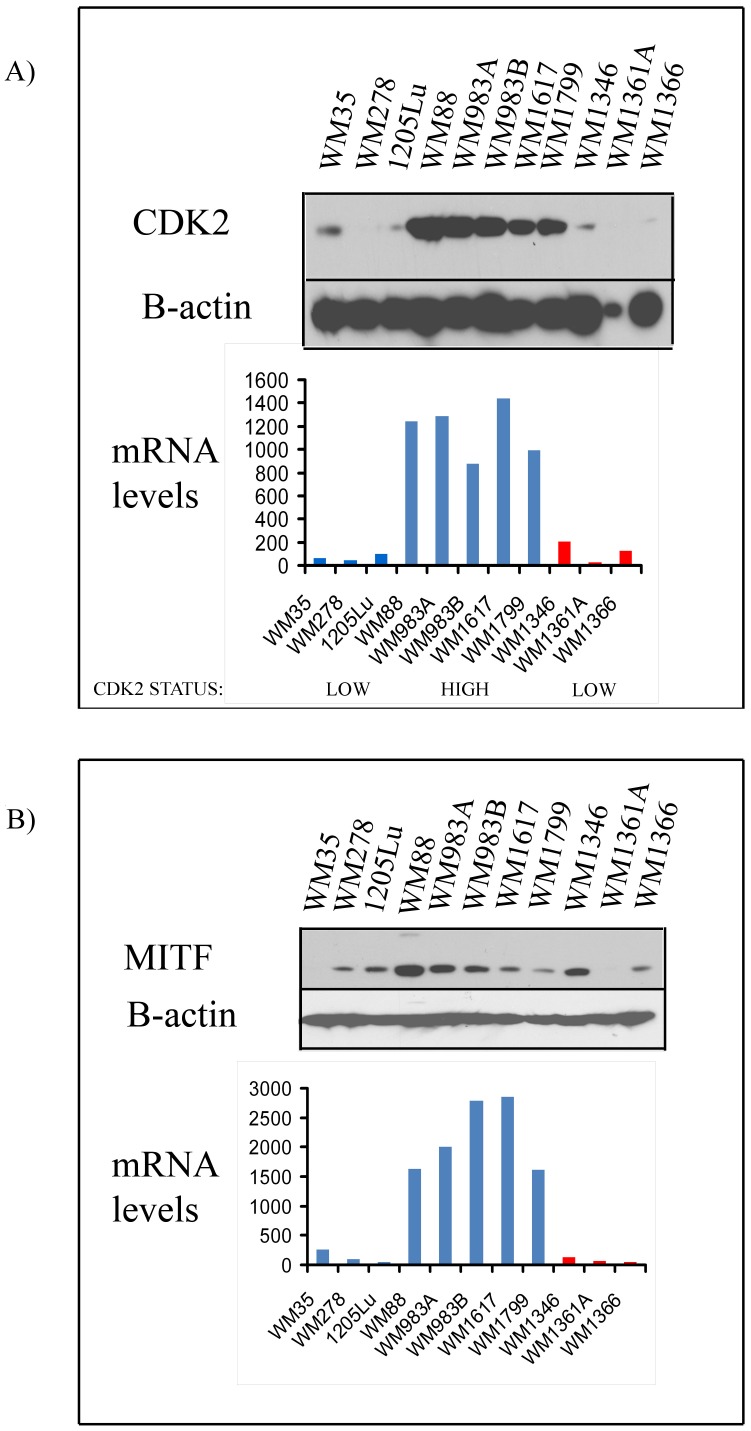
Expression of CDK2 and MITF in a panel of melanoma cell lines. A) CDK2 mRNA and protein expression levels in a panel of melanoma cell lines. mRNA levels in the melanoma panel were measured via microarray analysis. Data shows melanoma cell lines harboring the *BRAF* V600E mutation (blue) and the *NRAS* mutations (red). Protein expression was measured by Western blotting. B) MITF mRNA and protein expression levels in a panel of melanoma cell lines. mRNA levels in the melanoma cell lines were measured via microarray analysis. Data shows melanoma cell lines harboring the *BRAF* V600E mutation (blue) and the *NRAS* mutations (red). MITF protein expression was measured by Western blotting. Blots were stripped once and probed for actin to show equal protein loading. Relative CDK2 expression is denoted as “CDK2 LOW” and “CDK2 HIGH”.

Melanoma samples were prepared for analysis on the Affymetrix U133A array platform [27]. Cell lines represented in Fig. 1A and B are (in order): WM35, WM278, 1205Lu, WM88, WM983A, WM983B, WM1617, WM1799, WM1346, WM1361A, and WM1366. The data generated from these arrays has been published previously [27], [28] and have been deposited in the National Center for Biotechnology Information’s Gene Expression Omnibus website (http:/www.ncbi.nlm.nih.gov/geo/). Data are accessible using Gene Expression Omnibus Series accession GSE4845. Mutational profiling of *BRAF* (Exon 15) and *NRAS* (Exon 3) was performed by PCR as previously described [29].

### Adherent Cell Proliferation Assay

Cells were plated into a 96-well plate at a density of 2.5×10^4^ cells per mL and left to grow overnight. Cells were treated with increasing concentrations of dinaciclib in triplicate. In each experiment, cells were grown for 72 hrs treated with 20 µL 3-(4,5-dimethylthiazol-2-yl)-2,5-diphenyltetrazolium bromide (MTT; Sigma, St Louis, MO) reagent for 3 h. After this time, medium was rapidly removed and the resulting crystals were solubilized in DMSO. Absorbance was read in a plate reader at 540 nm. Absorbance readings were subtracted from the value of blank wells, and the reduction in cell growth was calculated as a percentage of control absorbance in the absence of any drug. Data show the mean of at least three independent experiments ± SE mean.

### Western Blot Analysis

Proteins were extracted and blotted as described in [25]. After analysis, Western blots were stripped once and re-probed for β-actin to show even protein loading. Antibodies for pRB ser807/811, caspase-3, Bcl-2, XIAP, and Mcl-1 were from Cell Signaling Technology (Beverly, MA). The monoclonal antibody to β-actin was from Sigma, the monoclonal antibody for CDK2 was from Santa Cruz (Santa Cruz, CA), and the p53 antibody was from Calbiochem (Darmstadt, Germany).

### Three-dimensional Spheroid Growth Assay

Melanoma spheroids were prepared using the liquid overlay method and treated as described previously [30]. Spheroids were treated with either 10 nM or 30 nM of dinaciclib for 72 hrs, prior to being stained using a LIVE/DEAD cell viability kit (Invitrogen, Carlsbad, Ca.).

### Cell Cycle Analysis

Cells were plated into 10 cm dishes at 60% confluency and left to grow overnight before treatment with 30 nM of dinaciclib for 12 or 24 hours. Cells were then treated and analyzed as described previously [13]. Briefly, melanoma cells were fixed in 70% ethanol and stained with propidium iodide. Samples were subsequently analyzed with an EPICS XL (Beckman-Coulter) apparatus.

### Flow Cytometric Analysis of Apoptosis

After treatment of the cells with 30 nM dinaciclib for 48 hr, cells were washed once with Annexin binding buffer [10 mmol/L HEPES (pH 7.4), 140 mmol/L NaCl, 5 mmol/L CaCl2], resuspended in 100 µL binding buffer containing 3 µL FITC-conjugated Annexin V (R&D Systems, Minneapolis, MN) and 1 µL Propidium Iodide (5 mg/mL Sigma Aldrich).

### In vivo Melanoma Xenograft Studies

Each treatment group consisted of five severe combined immunodeficient (SCID) CB-17 mice (Charles River Laboratories). Ten mice were injected subcutaneously (s.c.) with WM1366 cells (2×10^6^) in Matrigel into the lower flank. Once animals developed melanoma nodules of ∼5 mm in diameter, drug administration was initiated (day 1). SCID mice were randomly assigned to two experimental groups of five animals each: (*a*) 200 µL vehicle (20% hydroxypropyl β-cyclodextrin, HPBCD, Cargill Foods), and (*b*) 5 mg/kg dinaciclib in 200 µL HPBCD, three times per week intraperitoneally over a period of 14 days. Tumors were measured twice a week using digital calipers. Tumor volume was calculated as a product of three dimensions. Tumor shrinkage was calculated as a fold change relative to the starting volume. At treatment day 14, 1 h after the final drug application, all animals were euthanized.

### Statistical Analysis

Unless otherwise stated, all experiments show the mean ± SE mean of at least three independent experiments. Statistical significance was measured using Student’s *t* test, in which *P*<0.05 was judged to be significant.

## Results

### Dinaciclib Inhibits Melanoma Growth Independently of CDK2 Expression Levels and BRAF/NRAS Mutational Status in 2D and 3D Melanoma Models

It has been previously shown that MITF is critical for maintaining the expression of CDK2 in melanoma cells, and that that levels of both CDK2 and MITF predict responses to the CDK inhibitor roscotivine [24]. In an initial series of studies, we examined the relationship between CDK2/MITF mRNA and protein expression levels and response to treatment by dinaciclib. After screening a large panel of melanoma cell lines, we identified 5 melanoma cell lines that expressed high levels of CDK2 mRNA (CDK2 HIGH) and 6 that expressed low levels of CDK2 mRNA (CDK2 LOW) (Figure 1). As *BRAF/NRAS* mutational status is also thought to predict targeted therapy response in melanoma, we further sub-stratified the groups according to CDK2 melanoma cell lines that harbored mutations in either *BRAF* V600E or *NRAS*. The RNA levels of CDK2 correlated well with levels of CDK2 protein expression. In contrast, RNA levels of MITF were not found to clearly correlate with protein expression (Figure 1A–B).

**Figure 2:**
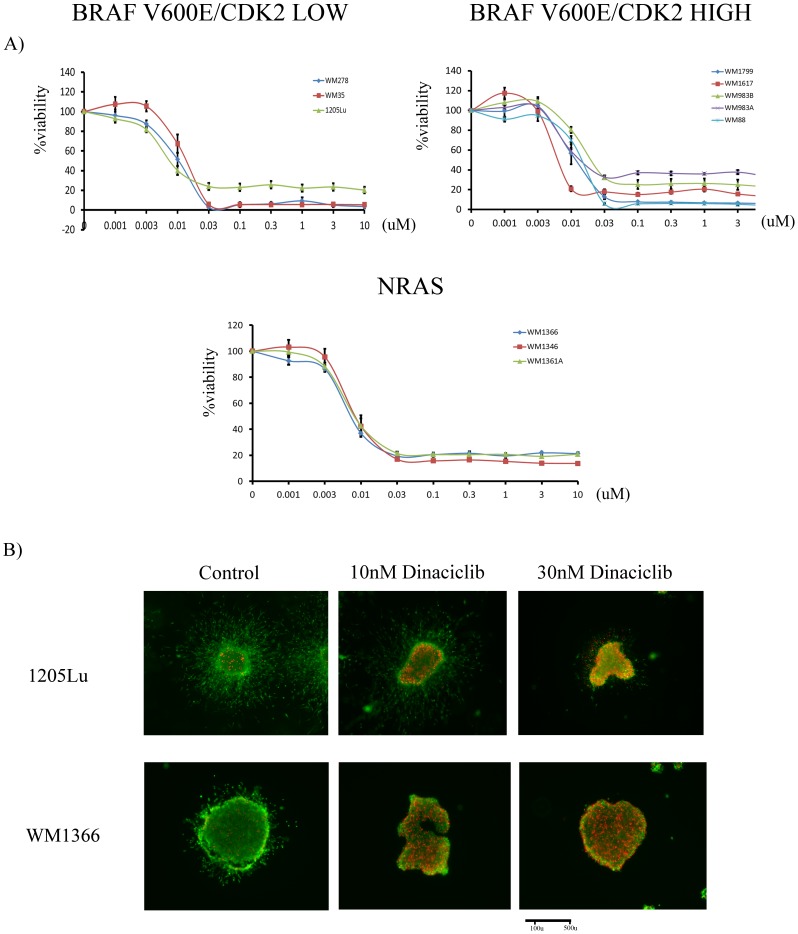
Dinaciclib inhibits the growth and survival of melanoma cell lines grown under both 2D adherent culture and 3D organotypic cell culture conditions. A) Growth inhibition for melanoma cells treated with dinaciclib measured by the MTT assay. Cells were treated with increasing concentrations of dinaciclib (1 nM–10 µM) for 72 hours before treatment with the MTT reagent. Absorbances were read at 570 nm and expressed as a percentage of control. B) Dinaciclib reduces the viability and invasion of melanoma cells grown as three-dimensional collagen-implanted spheroids. Preformed melanoma spheroids that harbored either the BRAF V600E mutation (1205Lu) or NRAS mutation (WM1366) were embedded into collagen and overlaid with medium. Cells were then treated with either 10 nM or 30 nM dinaciclib for 72 hours before being stained with calcein-AM and ethidium bromide. Spheroids were visualized and photographed using an inverted fluoresce microscope. *Green*, viable cells; *red*, dead cells.

Treatment of melanoma cells with increasing concentrations of dinaciclib for 72 h led to a concentration dependent inhibition of cell growth (Figure 2A). Interestingly, there was no selectivity in the extent of growth inhibition observed across the cell lines with either high/low CDK2 expression levels or the presence of a *BRAF/NRAS* mutation.

Previous studies from our group have shown that growth of melanoma cell lines as 3D collagen-implanted spheroids increases their drug resistance [30]. Here, we found that treatment of either the *BRAF* V600E mutated 1205Lu cell line or the *NRAS* mutated WM1366 cell line with dinaciclib for 72 hrs led to a marked loss of cell viability, as shown by the loss of green staining and increase in red staining, and lack of invasion into the surrounding collagen. The mean length of invasion was reduced from 160 µm in the control 1205Lu spheroids to 106 and 25 µm following treatment with dinaciclib at 10 nM or 30 nM respectively. Similarly, WM1366 spheroids treated with DMSO or dinaciclib at 10 nM or 30 nM displayed a length of invasion of 31, 5, and 3 µm respectively (Table S1).

### Dinaciclib Treatment is Associated with G2/M Phase Cell Cycle Arrest and the Induction of Apoptosis

**Figure 3:**
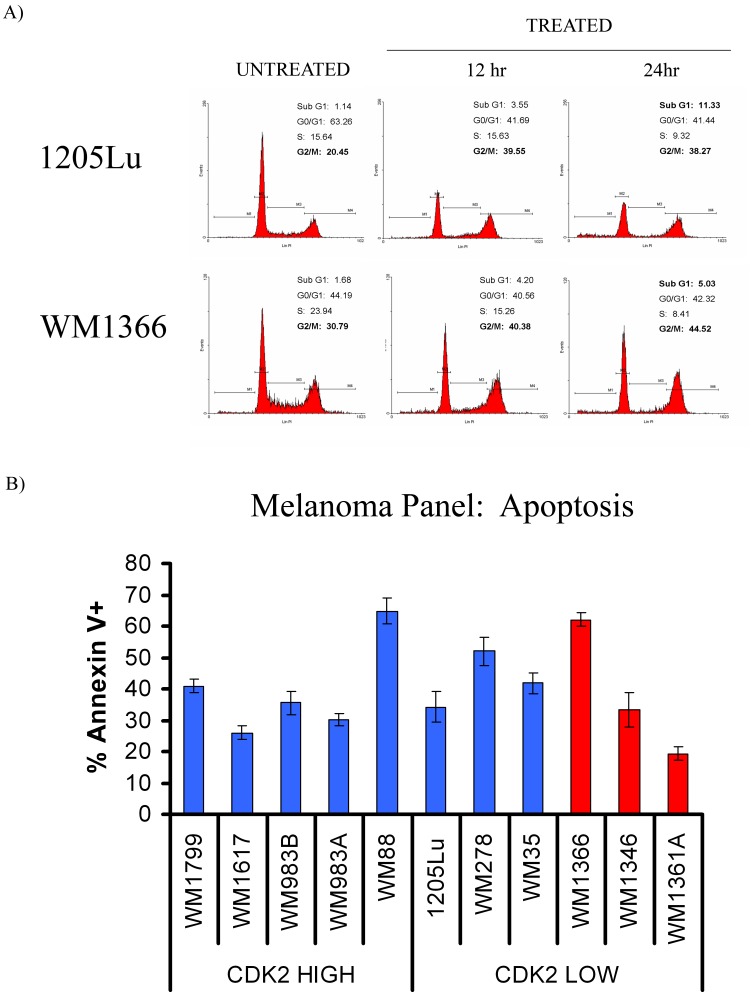
Dinaciclib causes a G2/M arrest and apoptosis in a panel of melanoma cell lines. A) Dinaciclib causes a G2/M arrest within 12 hours of treatment. Cell cycle analysis of a BRAF V600E (1205Lu) mutated or NRAS mutated (WM1366) cell line. Cells were treated with 30 nM of dinaciclib for either 12 hours or 24 hours; then cells were fixed for 24 hrs, and then stained for DNA content with propidium iodide. B) Dinaciclib causes apoptosis in a panel of melanoma cell lines independent of BRAF V600E *(blue),* NRAS *(red)* mutational status and CDK2 levels. Cells were treated with 30 nM dinaciclib for 48 hrs and apoptosis was assessed via Annexin-V staining. Data represent % of cells that were Annexin V +. Data shows mean of three independent experiments +/− S.E.

To examine the effect of dinaciclib on cell cycle progression, a *BRAF* V600E mutated cell line (1205Lu) and *NRAS* mutated cell line (WM1366) were incubated with 30 nM of drug for increasing periods of time (Figure 3A). Treatment of both cell lines with dinaciclib induced a G2/M phase cell cycle arrest associated with the loss of cells progressing through S-phase and an increase in the number of cells accumulating in the sub-G1 fraction of the cell cycle (Figure 3A). To further investigate the potential pro-apoptotic effects of dinaciclib and to examine if these effects were related to *NRAS/BRAF* mutational status or CDK2 expression levels, we treated the entire panel of cell lines with dinaciclib (30 nM) for 24 hrs (Figure 3B). Dinaciclib induced apoptosis in all cell lines tested, with effects ranging from 19% (WM1361A) to 65% (WM88) of Annexin V-positive cells (Figure 3B). The ability of dinaciclib to induce apoptosis in the melanoma cell line panel was unrelated to either CDK2 expression levels or *BRAF/NRAS* mutational status, suggesting that this drug had broad-spectrum anti-melanoma activity.

### Dinaciclib Causes Tumor Regression in a Mouse Xenograft Melanoma Model

**Figure 4:**
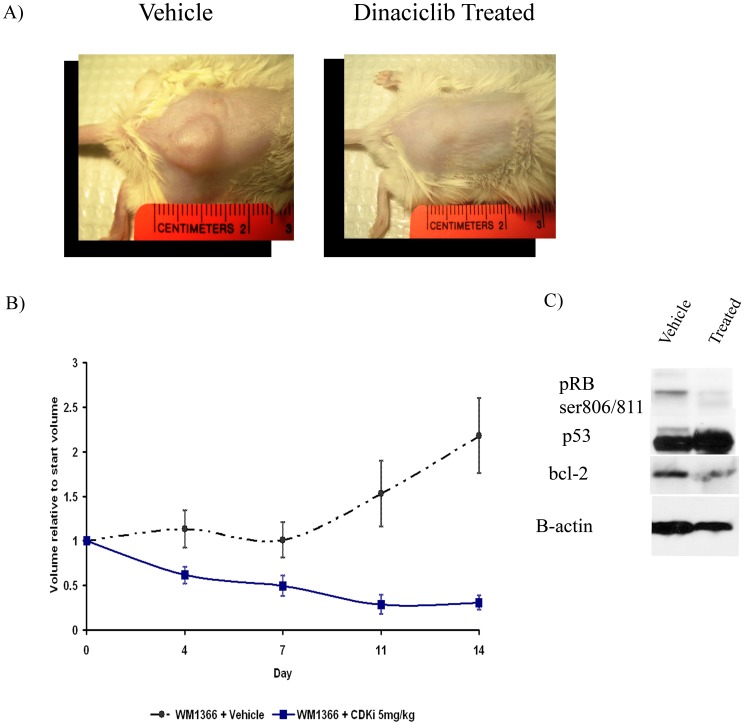
Dinaciclib induces regression of WM1366 melanoma xenografts. A) Photographs of representative vehicle and dinaciclib-treated WM1366 tumors taken after day 14 of treatment. After tumor establishment, mice were dosed twice daily with either vehicle (20% HPBCD) or dinaciclib (5 mg/kg in 20% HPBCD) intraperitoneally for 14 days. B) Growth curves of vehicle or dinaciclib-treated mice. Tumor volumes were measured twice a week and were normalized to the start volumes. Dinaciclib led to tumor regression after 14 days of treatment (p = .0046). C) Dinaciclib treatment led to the downregulation of pRBser807/811, upregulation of p53, and inhibition of Bcl-2 Tumor samples were extracted from SCID mice after 14 days of treatment with dinaciclib or vehicle and were analyzed via western blot. Blots were stripped once and equal protein loading was confirmed by probing the blots for actin expression.

We next examined whether the observed anti-melanoma effects of dinaciclib *in vitro* monolayer cultures and 3D organotypic models translated into the *in vivo* setting. As all of the melanoma lines tested responded to treatment in a similar manner in the *in vitro* assays, we chose to perform the mouse xenograft experiments with the WM1366 cell line (CDK2 low/*NRAS* mutated) to assess if the compound would still be effective if CDK2 levels were low. Furthermore, we were interested in determining if dinaciclib could have therapeutic efficacy in an NRAS mutant melanoma, as there are currently no effective therapies for these types of tumors. After tumor establishment (5×5 mm), SCID mice were dosed three times per week with 5 mg/kg dinaciclib by intraperitoneal injection. After 14 days, we observed that dinaciclib treatment significantly suppressed tumor growth, leading to regression of the original lesion (Figure 4A–B). Indeed, the tumor was barely visible following a two-week dinaciclib treatment (Figure 4A). To assess the biochemical effects of dinaciclib *in vivo*, tumors were harvested from both groups of mice at day 14 and were analyzed for markers of anti-CDK activity. Treatment of mice with dinaciclib was found to reduce RB phosphorylation at Ser807/811 (Figure 4C), indicative of CDK2 inhibition. We hypothesize that the dinaciclib pro-apoptotic effect was likely mediated by the inhibition of transcriptional CDKs such as CDK7 and CDK9, which regulate the expression of anti-apoptotic proteins such as Bcl-2 and Mcl-1. In line with this hypothesis, we found a decrease in Bcl-2 expression in the dinaciclib treated tumors and an increase in p53 expression (Figure 4C). Sequencing of p53 in the WM1366 cell line indicated a p53 E285K missense mutation in the transactivation domain. This mutant has a nearly identical transactivation activity compared to wild type p53 [31].

### Dinaciclib Down-regulates Retinoblastoma Phosphorylation and the Expression of Anti-apoptotic Proteins in vitro

**Figure 5:**
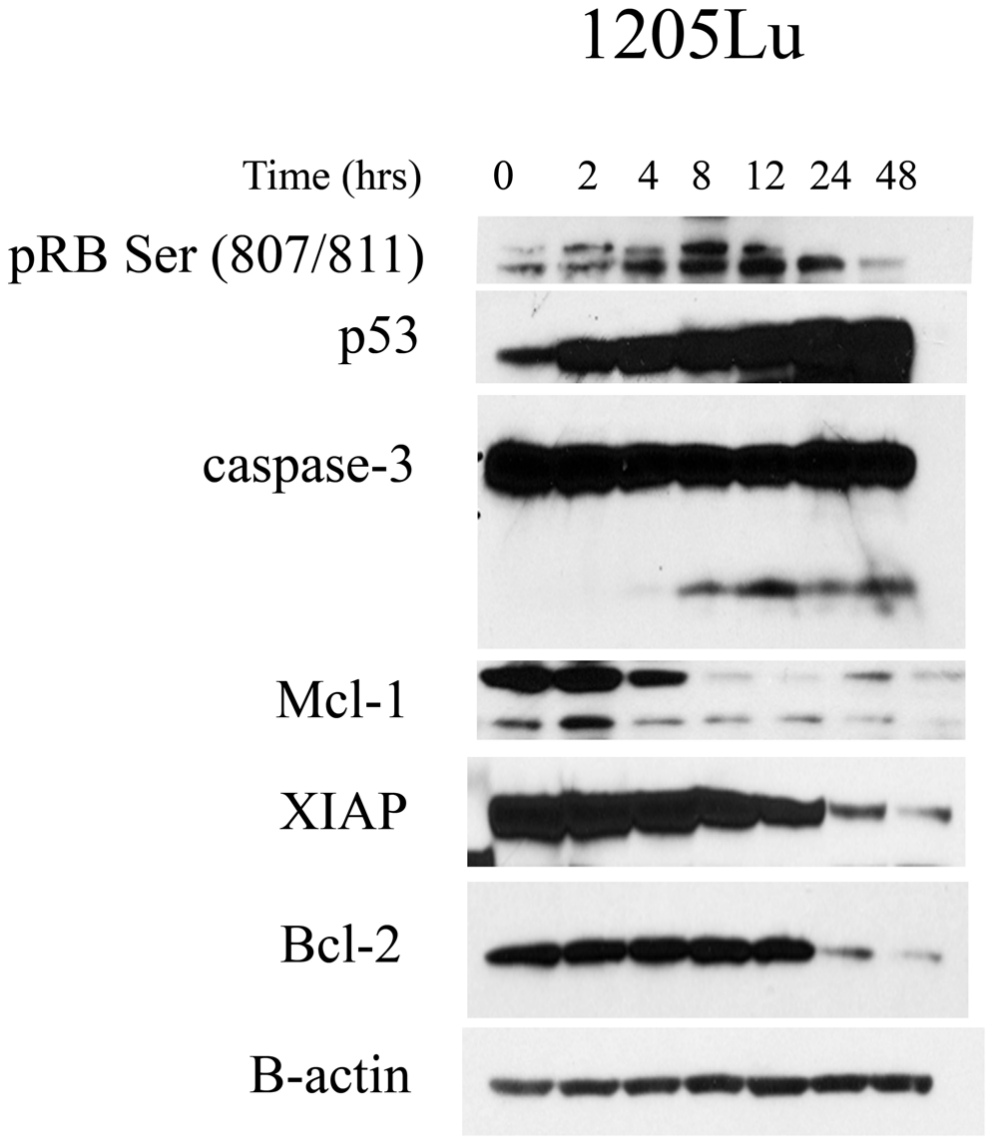
Dinaciclib treatment induces the up-regulation of p53 expression and caspase-3 cleavage and a downregulation of the pro-survival molecules Bcl-2, XIAP, and Mcl-1 in vitro. Western Blot data of dinaciclib treated 1205Lu cells treated with 30 nM of dinaciclib for increasing periods of time (0–48 hrs). Along with a decrease in pRBser807/811, dinaciclib induced a marked upregulation of p53, increase in cleaved caspase-3, and down regulation of the pro-survival molecules Bcl-2, XIAP, and Mcl-1. The membrane was probed for actin expression to ensure equal protein loading.

Next, we confirmed the biochemical data from our *in vivo* study in a series of *in vitro* experiments. Treatment of the *BRAF* V600E-mutated melanoma cell line 1205Lu with dinaciclib (30 nM, 0–48 hrs) led to a decrease in the extent of RB phosphorylation at Ser 807/811(Figure 5). At the same time, we noted that the level of p53 expression markedly increased. Interestingly, the increase in p53 occurred more rapidly than the observed decrease in RB phosphorylation. Consistent with the pro-apoptotic effects of dinaciclib and the increased expression of p53, we noted a rapid induction of caspase-3 cleavage and a decrease in the anti-apoptotic proteins Mcl-1, XIAP, and Bcl-2 (Figure 5). Similarly, when the WM35 cell line was treated with dinaciclib 30 nM RB phosphorylation at Ser 807/811 decreased and p53 increased. The pro-apoptotic effect of dinaciclib could be observed by an increase in PARP cleavage (Figure S1).

### Dinaciclib Induces p53 Dependent Apoptosis in Melanoma Cells

**Figure 6:**
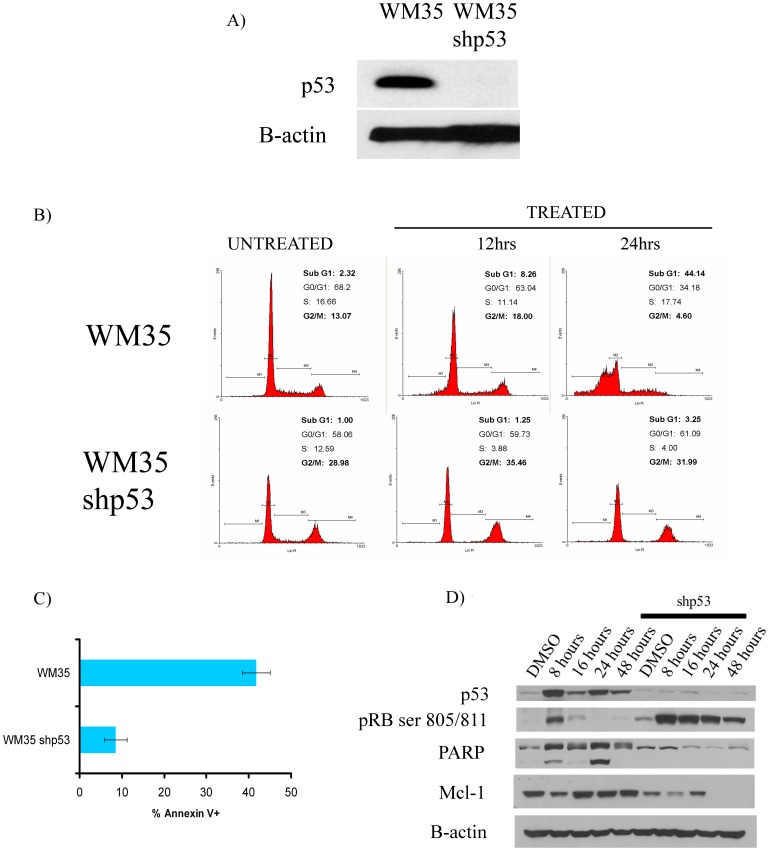
shRNA knockdown of p53 expression reverses dinaciclib-induced apoptosis. A) Knockdown of p53 levels using a lentiviral shRNA construct. WM35 melanoma cells were infected with a lentivirus encoding for shRNA against p53 and were selected by flow cytometric sorting for GFP (designated WM35 shp53). Western blot shows knockdown of p53 protein. Actin shows equal protein loading. B) WM35 cells expressing p53 shRNA show diminished cell accumulation in the sub-G1 phase of the cell cycle following dinaciclib treatment. Cells were treated with 30 nM of dinaciclib for either 12 hours or 24 hours, before being fixed and stained for DNA content with propidium iodide. C) Apoptosis in a wild-type p53 melanoma cell line is dependent upon p53. WM35 and WM35 shp53 cells were treated with 30 nM dinaciclib for 48 hrs and were assessed for apoptosis via Annexin-V staining. WM35 shp53 cells exhibited a dramatic reduction of apoptosis when compared to WM35. D) The knockdown of p53 led to decreased pRB (ser807/811) and a decrease in cleaved PARP in cells treated with dinaciclib. WM35 and WM35 shp53 cells were treated with 30 nM of dinaciclib for 8, 16, 24, and 48 hrs, after which protein were assessed for the levels of p53, pRBser807/811, PARP, and Mcl-1. Actin was used as loading control.

Although most melanomas lack p53 mutations, they are typically poor at undergoing p53-dependent apoptosis. As dinaciclib induced p53 expression and was strongly pro-apoptotic, we next determined the role of p53 induction in dinaciclib-induced apoptosis. In an initial series of experiments, we generated two melanoma cell lines in which p53 expression was stably knocked down (>90%) using a lentiviral shRNA vector (Figure 6A and Figure S2). Treatment of cells expressing p53 shRNA with dinaciclib was associated with decreased apoptosis, evidenced by a lack of accumulation of cells in the sub-G1 phase of the cell cycle (Figure 6B). Although little apoptosis was seen, some cell cycle arrest was still observed in the p53 knockdown cells, associated with a progressive loss of cells proceeding through S-phase (Figure 6B). Consistent with these results, it was also noted that the level of apoptosis as measured by FITC-annexin-V was also significantly attenuated, p-value (p<.001) (Figure 6C), further demonstrating the role of increased p53 expression in the pro-apoptotic effects of dinaciclib. Mechanistically, the p53 knockdown cells also exhibited a decrease in RB ser807/811 phosphorylation and lack of PARP cleavage following dinaciclib treatment relative to the control cell lines (Figure 6D and Figure S2). Although the p53 knockdown cells exhibited much reduced apoptosis, dinaciclib was still able to reduce the expression of the anti-apoptotic protein Mcl-1 in a similar manner to that seen in the control WM35 cells (Figure 6D).

## Discussion

Overcoming intrinsic and acquired drug resistance of melanoma is a major challenge. Melanomas are relatively unique in cancer biology in being intrinsically resistant to nearly every chemo-therapeutic agent tried thus far [11]. Novel therapies targeting the BRAF oncogene, which is mutated in almost 50% of all melanomas, have demonstrated clinical benefit in melanoma patients. However, repeated experience has shown that melanomas acquired resistance even to new agents selectively targeting BRAF [4]. In this study, we characterize an inhibitor of CDKs with remarkable pro-apoptotic activity against human melanoma cells grown under 2D adherent culture conditions, 3D organotypic culture conditions, and in mouse xenografts.

There is currently much interest in the idea of “personalized cancer therapy”, a paradigm whereby patients are selected based on the genetic/mutational profiles of their tumors and given targeted therapies directed against the driving oncogenic mutations. This approach has shown success in melanoma, where carefully selected patients harboring BRAFV600E activating mutations are receiving inhibitors of the MAPK pathway, which is constitutively active due to this mutation [5], [32]. This approach is also self-evident when considering CDK’s as a potential melanoma-specific therapeutic target. Previous work has shown that melanoma sub-groups exist with either high or low expression levels of CDK2, with the low CDK2 expressing cell lines posited to be the optimal group for receiving CDK2 inhibitor therapy [24]. An initial screen of our panel of melanoma cell lines identified a group of melanoma cell lines with high CDK2 expression and a group with low CDK2 expression. At the RNA level, there was good positive correlation between levels of CDK2 and the transcription factor MITF in both *BRAF-V600E* and *NRAS* mutated melanoma cell lines. Interestingly, there was also some stratification between CDK2 expression level and *BRAF/NRAS* mutational status. We also found that all of the *NRAS* mutated melanoma lines evaluated (or included in our panel) had low expression of CDK2, whereas there was some variability within the *BRAF*-V600E mutated group, with some cell lines harboring high expression of CDK2 and some with low expression. These results are consistent with CDK2 being a downstream target of the MITF transcription factor in melanoma [24], as previous work has shown that all of the cell lines in the NCI-60 cell line panel with MITF amplification also harbored a *BRAF* V600E mutation [33]. Although we saw agreement between CDK2 and MITF levels at the RNA level, there was some variability in the levels of MITF protein expression that did not always match with CDK2 protein levels. It is likely that this lack of clear correlation is a consequence of the complex system of post-translational modification and proteasomal targeting seen with MITF.

The observed anti-melanoma effects most likely result from cdk inhibition, though secondary effects of the compound cannot be ruled out.

Treatment of a panel of melanoma cell line with dinaciclib led to a very potent inhibition of cell growth and induction of apoptosis. There was no correlation between CDK2 expression levels and sensitivity of the cell lines to dinaciclib, suggesting that dinaciclib is not functioning exclusively as a CDK2 inhibitor. As it has been previously shown that the *BRAF* V600E mutation conveys sensitivity of melanoma cells to BRAF [34], [35], [36] and MEK [37] inhibitors and the phenothiazine compounds [38], we next addressed whether the presence of a *BRAF* or *NRAS* mutation conferred sensitivity to dinaciclib. We observed that there was no apparent correlation between dinaciclib response and *BRAF/NRAS* mutational status, leading us to conclude that dinaciclib had broad-spectrum anti-melanoma activity and was not conclusively mutation specific.

A number of previous studies have shown that intracellular signaling and drug resistance are modified by the tumor microenvironment. In particular, our group has previously shown that melanoma cells derived from metastases become almost completely resistant to certain inhibitors when grown under 3D organotypic cell culture conditions [30]. We found that both the *BRAF*-mutated 1205Lu and *NRAS*-mutated WM1366 melanoma cell lines retain their sensitivity to dinaciclib when grown as 3D collagen-implanted spheroids, suggesting that modulating the tumor microenvironmental conditions does not increase resistance to this compound. Other melanoma targeted therapies, such as the MEK inhibitors, are associated with reversible cytostatic effects when grown under 3D organotypic cell culture and *in vivo* conditions [39]. Here we show that a 14-day dinaciclib treatment of established WM1366 melanoma tumors *in vivo* led to a marked inhibition of tumor growth and some degree of regression, suggesting that dinaciclib was having significant cytotoxic effects beyond induction of G2/M phase cell cycle arrest. Biochemically, dinaciclib treatment led to reduced expression of the anti-apoptotic proteins Bcl-2, Mcl-1 and XIAP. Downregulation of these anti-apoptotic proteins is a common feature of other CDK inhibitors, particularly those that have selectivity for CDK9 [15]. Thus it has been shown that both UCN-01 and flavopiridol decrease the expression of Mcl-1, XIAP, BAG-1, and Bcl-2 in B-cell chronic lymphocytic leukemia cells [15], [40]. The downregulation of pro-apoptotic gene expression following CDK9 inhibition is likely to be one of the major pro-apoptotic mechanisms of these compounds. In particular, the ability of dinaciclib to regulate these anti-apoptotic proteins is likely to be of great utility in melanoma where overexpression of XIAP [41], Bcl-2 [42] and Mcl-1 [43] is commonplace and can contribute to melanoma chemoresistance.

Dinaciclib treatment also led to the induction of p53 expression under both *in vitro* and *in vivo* conditions. The role of p53 in melanoma and its potential role in therapy remains somewhat unclear since melanomas generally harbor very low rates of p53 mutations and are generally poor at undergoing p53-dependent apoptosis. Previous work from our group has shown that melanomas can tolerate high levels of p53 expression and do not undergo apoptosis [13]. The lack of p53-dependent apoptosis under these conditions is likely a consequence of high expression levels of the E3-ubiquitin ligase MDM2 (or HDM2) [44], which holds the transcriptional activity of p53 in check by targeting it to the proteasome. More than 50% of primary invasive and metastatic melanomas overexpress HDM2 which contributes to the lack of p53 activity in melanoma [44]. More recently, it has been shown that MDM4 is upregulated in 65% of melanomas promoting tumor survival by counteracting the effects of p53 [45]. However, under certain conditions, p53 can become pharmacologically activated in melanoma cells. We and others have previously demonstrated that the pharmacological activation of p53 via either GSK3β inhibition or Nutlin-3 induced high levels of apoptosis in melanoma cells [13], [46]. Activation of p53 also seems to be a critical determinant of dinaciclib-induced apoptosis, with selective knockdown of p53 expression leading to a marked inhibition of dinaciclib-induced apoptosis. Interestingly, knockdown of p53 expression was still associated with the dinaciclib-induced reduction in the expression of Mcl-1. This demonstrates either that loss of Mcl-1 was not critical to the pro-apoptotic activity of dinaciclib or that the reduced expression of Mcl-1 still requires functional p53 to induce apoptosis.

Dinaciclib has shown preclinical in vitro and in vivo activity in pancreatic, osteosarcoma and melanoma models [47], [48], [49]. Dinaciclib showed moderate activity in a phase 2 trial of melanoma with acceptable toxicity [50]. Our work mechanistically supports the anti-melanoma activity of dinaciclib and further demonstrates its potential as a novel therapy for this disease. In addition, the ability of dinaciclib to pharmacologically activate p53 suggests that dinaciclib could be an ideal candidate for combinations with cytotoxic chemotherapies, as a possible mechanism to overcome chemoresistance. This idea fits well with previously published studies showing that flavopiridol potentiates the induction of apoptosis seen following treatment with mitomycin C, paclitaxel, SN-38, and topoisomerase I inhibitors in gastric and breast cancer cell lines [51]. In these studies, p53 function was critical for the flavopiridol’s ability to potentiate chemotherapy-induced apoptosis, with the extent of apoptosis induced being 5-fold greater in p53 (+/+) HCT116 cells compared to that seen in p53 (−/−) HCT166 cells [51]. Since most melanomas lack p53 mutations [3], it can be reasoned that dinaciclib should have good pro-apoptotic activity across the spectrum of melanoma patients when combined with agents such as those targeted against BRAF/MEK signaling. Data have previously shown that melanoma cells do not undergo apoptosis following MEK inhibitor treatment as a result of constitutively high Mcl-1 expression levels [43]. A compound such as dinaciclib, which downregulates Mcl-1 expression, could be an ideal combination candidate in future preclinical and clinical studies.

## Supporting Information

Figure S1 **Western Blot data of WM35 cells treated with 30 nM of dinaciclib for increasing periods of time (0–48 hrs).** Along with a decrease in pRBser807/811, dinaciclib induced a marked upregulation of p53, and increase in cleaved PARP. Actin was used to ensure equal protein loading.
(TIF)

Figure S2 **Knockdown of p53 levels using a lentiviral shRNA construct.** 1205Lu melanoma cells were infected with a lentivirus encoding for shRNA against p53 and were selected by flow cytometric sorting for GFP (designated 1205Lu shp53). 1205Lu and 1205Lu shp53 cells were treated with 30 nM of dinaciclib for 8, 16, 24, and 48 hrs, Western blot shows knockdown of p53 protein. 1205Lu cells treated with dinaclib showed decrease in pRB (ser807/811) and increase in cleaved PARP as an indicator of apoptosis. In The 1205Lu shp53 cells dinaciclib treatment did not induce cleavage of PARP and only a slight decrease in pRB levels. Actin was used as loading control.
(TIF)

Table S1 **Quantification of spheroid invasion into surrounding collagen matrix.** Preformed melanoma spheroids that harbored either the BRAF V600E mutation (1205Lu) or NRAS mutation (WM1366) were embedded into collagen and overlaid with medium. Spheroids were then treated with either 10 nM or 30 nM dinaciclib for 72 hours before spheroids were visualized and photographed using an inverted fluorescence microscope. Images were analyzed for invasive length with a maximum number of 80 samples analyzed per image. The mean length in µm and standard error of the mean are shown.
(DOCX)
