# Supplementary figures and images for: The Anti-Melanoma Activity of Dinaciclib, a Cyclin-Dependent Kinase Inhibitor, Is Dependent on p53 Signaling
Source: PLoS One. 2013 Mar 18;8(3):e59588. doi: 10.1371/journal.pone.0059588 (PMC3601112; doi:10.1371/journal.pone.0059588)

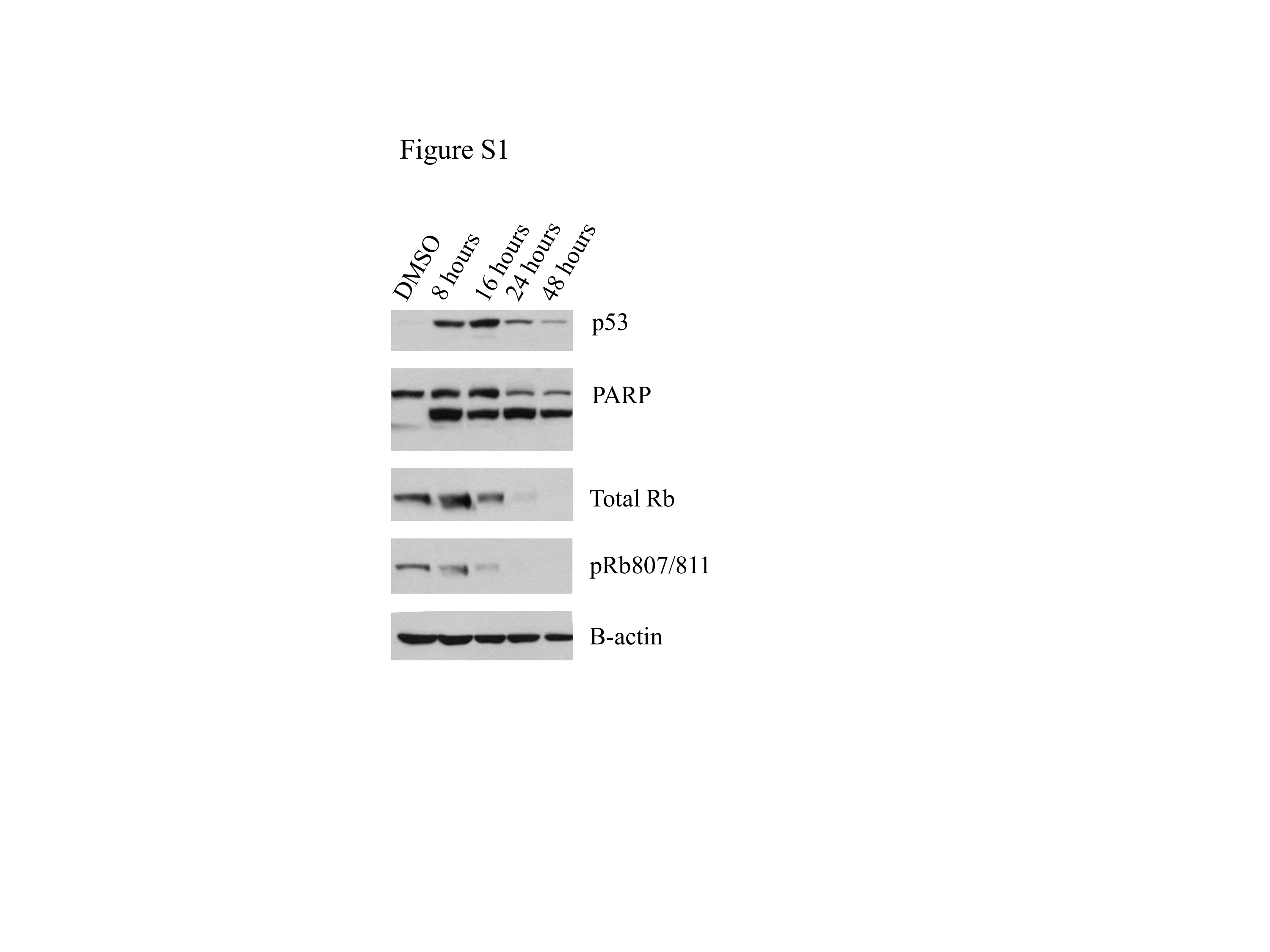

Supplement: Figure S1 — Western Blot data of WM35 cells treated with 30 nM of dinaciclib for increasing periods of time (0–48 hrs). Along with a decrease in pRBser807/811, dinaciclib induced a marked upregulation of p53, and increase in cleaved PARP. Actin was used to ensure equal protein loading. (TIF) [file pone.0059588.s001.tif]
