# Supplementary material for: The Anti-Melanoma Activity of Dinaciclib, a Cyclin-Dependent Kinase Inhibitor, Is Dependent on p53 Signaling
Source: PLoS One. 2013 Mar 18;8(3):e59588. doi: 10.1371/journal.pone.0059588 (PMC3601112; doi:10.1371/journal.pone.0059588)
Supplement: Table S1 — Quantification of spheroid invasion into surrounding collagen matrix. Preformed melanoma spheroids that harbored either the BRAF V600E mutation (1205Lu) or NRAS mutation (WM1366) were embedded into collagen and overlaid with medium. Spheroids were then treated with either 10 nM or 30 nM dinaciclib for 72 hours before spheroids were visualized and photographed using an inverted fluorescence microscope. Images were analyzed for invasive length with a maximum number of 80 samples analyzed per image. The mean length in µm and standard error of the mean are shown. (DOCX) [file pone.0059588.s003.docx]

|  |  |  |  |  |
| --- | --- | --- | --- | --- |
|  |  | sample number | mean length, μm | SEM |
| 1205Lu | control | 80 | 160.0 | 4.7 |
| 1205Lu | dinaciclib 10nM | 80 | 106.3 | 4.1 |
| 1205Lu | dinaciclib 30nM | 67 | 24.7 | 1.2 |
| WM1366 | control | 74 | 31.0 | 2.9 |
| WM1366 | dinaciclib 10nM | 13 | 5.0 | 0.9 |
| WM1366 | dinaciclib 30nM | 9 | 3.2 | 1.4 |

**Table S1**
